# The impact of diabetic polyneuropathy on toe grip strength in patients with type 2 diabetes mellitus: a cross-sectional study

**DOI:** 10.1007/s40200-023-01260-y

**Published:** 2023-08-02

**Authors:** Koji Yoshida, Yasushi Miura, Shuhei Nakanishi, Shigeharu Tanaka, Katsushi Kuniyasu, Shinsuke Matsumoto, Kozo Hanayama

**Affiliations:** 1 Department of Rehabilitation Science, Kobe University Graduate School of Health Sciences, Kobe, Japan; 2 Department of Rehabilitation Center, Kawasaki Medical School Hospital, Kurashiki, Japan; 3 Division of Diabetes, Metabolism and Endocrinology, Kawasaki Medical School, Kurashiki, Japan; 4 School of Rehabilitation, Kanagawa University of Human Services, Yokosuka, Japan; 5 Department of Physical Therapist, Faculty of Rehabilitation, Kawasaki University of Medical Welfare, Kurashiki, Japan; 6 Department of Physical Therapist, Kawasaki Junior College of Rehabilitation, Kurashiki, Japan; 7 Department of Rehabilitation Medicine, Kawasaki Medical School, Kurashiki, Japan

**Keywords:** Toe grip strength, Diabetes mellitus, Diabetic polyneuropathy, Foot care

## Abstract

**Purpose:**

Type 2 diabetes mellitus (T2DM) and concomitant diabetic polyneuropathy (DPN) induce muscle weakness. Muscle weakness in the foot is associated with foot deformities and falls. However, factors affecting toe grip strength (TGS) are not well known. Therefore, the present study investigated factors related to TGS in patients with T2DM.

**Methods:**

This was a cross-sectional study involving 100 patients with T2DM who were hospitalized for the treatment of T2DM and 50 healthy adults. The subjects were divided into three groups: a group of healthy subjects, a group of T2DM patients without DPN, and a group of T2DM patients with DPN. Hierarchical multiple regression analysis was performed with TGS and the TGS-to-weight ratio (TGS/Wt%) as dependent variables and with age, the presence of T2DM, and DPN as independent variables, and sex and BMI as confounders.

**Results:**

There were no significant differences in age or sex among the three groups. In the final regression analysis, age and presence of T2DM and DPN were associated in both models with TGS and TGS/Wt% as dependent variables.

**Conclusion:**

DPN, T2DM, and age were found to be related to TGS. The findings of this study could contribute to healthcare providers developing foot care and rehabilitation programs for diabetic patients.

**Trial registration:**

This study was registered with UMIN-CTR (UMIN000034320) on 1 November 2018.

## Background

The prevalence of diabetes mellitus (DM) is increasing worldwide, with one in ten adults estimated to have DM. The global population with DM is projected to grow to 783 million by 2045 [1]. Falls are also a major concern for older people with DM. In a longitudinal aging study, 31.6% of older individuals with DM experienced falls, whereas 19.4% of those without DM did [2]. Although DM on its own is a risk factor for falls, a multivariate analysis has reported an increased risk of falls with concomitant diabetic neuropathy (DPN) [3].

The weakness of lower limb muscle strength has been investigated as a factor in falls. A systematic review of falls reported that, in older people, lower limb muscle weakness is associated with a 1.76-fold increase in the likelihood of falling [4]. Lower limb muscle weakness in patients with DM is observed regardless of age, with knee extensor strength being 10–20% lower in patients with DM than in individuals without DM [5]. In addition, DPN causes knee extensor muscle weakness, particularly in middle-aged and older patients [6]. DPN commonly presents as a distally symmetric sensorimotor polyneuropathy [7]. More often, patients with DM show more distal lower limb muscle weakness than proximal lower limb weakness [8]. Andersen et al. reported that foot muscle volume was reduced by up to 50% in patients with DPN compared to patients without DPN [9]. Distal lower limb toe muscle strength plays a role in maintaining standing posture and controlling gait, and reduced toe grip strength (TGS) is a risk factor for falls [10–12]. Thus, accurate assessment of toe muscle strength is necessary to prevent falls in patients with DM.

Several preceding studies reported the factors affecting toe muscle strength in patients with DM. These studies assessed toe muscle strength in patients with DM by using the Paper Grip Test as a screening test and dynamometry [13–15] and found the factors affecting toe muscle strength, including the presence of DPN. The diagnostic criteria of the Toronto Expert Panel on Diabetic Neuropathy [16] and the simple diagnostic criteria proposed by the Japanese Study Group on Diabetic Neuropathy [17] are widely used to assess DPN. However, these criteria are simplified diagnostic criteria using subjective symptoms and physical findings and have been noted to have limitations relating to reliability and accuracy in diagnosing DPN [18]. Therefore, more objective testing to clarify the relationship between TGS and DPN is desired. To the best of our knowledge, there are no reports investigating the relationship between nerve conduction testing and toe muscle strength to establish the reliability of quantitative pathological and physiological tests for diagnosing DPN. In addition, no analysis has considered the confounding effect of sex and diagnosis of DPN by nerve conduction studies [6] on muscle strength in patients with DM. Therefore, it remains unclear which factors influence TGS the most. This study aimed to investigate the factors affecting TGS and further clarify the association with DPN based on quantitative nerve conduction studies using the Baba classification [19].

## Methods

### Study design and population

This cross-sectional study was conducted between December 2018 and February 2022. The study involved 100 inpatients diagnosed with type 2 DM (T2DM) at a hospital in Kurashiki City, Japan. All patients were admitted to the hospital because they had difficulty controlling their hyperglycemia and managing their lifestyles, including nutrition and exercise, and needed help. The 50 healthy subjects were volunteers recruited from Kawasaki Medical School Hospital and community residents in Tottori and Okayama prefectures for physical fitness measurement. The inclusion criteria were people aged 20 years and over who could walk 10 m independently without using a walking aid. The study exclusion criteria were unable to understand the study description, inability to walk unaided, and the presence of foot ulcers. These factors were set as exclusion criteria because they affect the participants’ safety and the data’s accuracy.

### Ethics

The study complied with the principles of the Declaration of Helsinki and was approved by the authors’ institutional ethics review board (number 3256-01). All participants provided written, informed consent to participate in the study. The study was registered with UMIN-CTR (UMIN000034320).

### Dependent variable

TGS was measured using a toe grip dynamometer (Takei Scientific Instruments, Niigata, Japan). Subjects sat upright on a chair without leaning on the backrest position with the hip and knee joints flexed about 90° and the neutral ankle position for 3 s maximal voluntary contractions. The mean TGS and the TGS-to-weight ratio (TGS/Wt%) were calculated from the measured TGS values. The reliability of the TGS has been confirmed in previous studies [20, 21].

### Independent variable

Data on age, sex, body mass index (BMI), duration of T2DM, ankle-brachial index (ABI), HbA1c, sensory examination, and history of falls in the past six months were collected. Walking speed and grip strength were measured using the Asian Working Group for Sarcopenia Guidelines Methods [22, 23]. Grip strength was measured once for each hand using a hand grip dynamometer (Takei Scientific Instruments, Niigata, Japan) in an end-sitting position with elbow extension, and the larger value of both sides was used. Walking speed was assessed using the 4-m walking speed test at a comfortable walking speed on a flat surface. Four sensory tests were performed on patients with T2DM. Loss of protective sensation (LOPS) and vibration perception were assessed according to the International Working Group on the Diabetic Foot Practical Guidelines [24]. LOPS was evaluated using the Semmes-Weinstein 10-g monofilament and vibration perception using a 128-Hz tuning fork. The patients were interviewed to determine whether they were aware of any lower limb neuropathy. Nerve conduction studies were performed by an experienced neurologist. This study used the Baba classification of severity [25], which classifies the severity of DPN into five stages according to nerve conduction studies in the lower limb: stage 0 is normal without abnormal nerve conduction studies, and stage 5 is the most severe DPN. In this study, patients with T2DM were classified into two groups based on the Baba classification: without DPN group (without DPN) and with DPN group (with DPN). A dummy variable was coded as 0 without DPN and 1 with DPN.

### Sample size calculation

The α value was set at 0.05, and the power was set at 0.80. Based on a previous study [21], the hypothesis explanatory variance adjusted for degrees of freedom was 0.31, and a total of 48 cases were estimated to be needed for the five potential variables in this study.

### Statistical analysis

The subjects were divided into three groups (healthy subjects, patients without DPN, and patients with DPN) for comparisons among the three groups. The Shapiro-Wilk test was performed to confirm the normality of each variable. One-way analysis of variance, the Kruskal-Wallis test, and Tukey’s post hoc test were performed for continuous variables. The χ^2^ test and Holm’s post hoc test were used for categorical variables. Hierarchical multiple regression analysis was performed to identify factors associated with TGS. Four models were constructed in this study with TGS and TGS/Wt% as dependent variables. First, independent variables such as age, T2DM, and DPN were entered into the model using forced entry. Second, sex and BMI were forced into the regression model as confounding factors in model 2 with TGS as the dependent variable. Sex was entered into model 4 as a confounding factor with TGS/Wt% as the dependent variable. All data were analyzed using SPSS (version 26.0, SPSS Inc, Chicago, IL, USA) or EZR (version 1.55, Saitama, Japan). Values of p < .05 were considered to indicate significance.

## Results

There were 150 participants in the study. Two T2DM patients without nerve conduction studies were excluded, and 148 patients were included in the analysis. There were no significant differences in age or sex among the three groups: healthy subjects, patients without DPN, and patients with DPN. Weight and BMI were significantly higher in the patients without DPN and the patients with DPN than in the healthy subjects (Table 1). Table 2 shows the results of group comparisons of physical function and history of falls in the healthy subjects, the patients without DPN, and the patients with DPN. TGS and TGS/Wt% were significantly lower in the patients without DPN and the patients with DPN than in the healthy subjects, and significantly lower in patients with DPN than in patients without DPN. In the healthy subjects, none had abnormalities in LOS or VPT, although the Baba classification was not applicable. In terms of the history of falls, patients with DPN had more histories of falls than the healthy group, although the patients without DPN did not.

**Table 1:** Participants’ characteristics

	healthy subjects	patients without DPN	patients with DPN	*P* value
n	50	50	48	
Age, years	60.4 (16.5)	61.2(10.7)	64.0(11.5)	0.477
Sex (women), n (%)	29 (58.0)	22 (44.0)	18 (37.5)	0.114
Height, cm	159.6 (10.4)	161.9 (8.9)	162.9 (8.4)	0.204
Weight, kg	56.6 (10.6)	68.7 (15.1)^a^	66.3 (13.7)^a^	< 0.001
BMI, kg/m^2^	22.1 (2.6)	26.1(4.8)^a^	25.0(5.0)^a^	< 0.001
HbA1c, %	-	9.8 (1.7)	10.4 (2.7)	0.328
Duration of diabetes, year	-	10.5 (8.1)	15.2 (11.4)	0.055
Nephropathy, n (%)	0 (0.0)	45 (90.0)^a^	46 (95.8)^a^	< 0.001
Baba grade, n (%)				
0	-	50 (100)	0 (0.0)	< 0.001
1	-	0	23 (47.9)	
2	-	0	18 (37.5)	
3	-	0	6 (12.5)	
4	-	0	1 (2.1)	
Retinopathy, n (%)	0 (0.0)	8 (16.3)^a^	21 (43.8)^ab^	< 0.001
ASO, n (%)	0 (0.0)	2 (3.9)	3 (6.2)	0.221
Hypertension, n (%)	11 (22.0)	33 (66.0)^a^	30 (62.5)^a^	< 0.001
Hyperlipidemia, n (%)	1 (2.0)	40 (80.0)^a^	36 (75.0)^a^	< 0.001
Heart disease, n (%)	0 (0.0)	7 (14.0)^a^	12 (25.0)^a^	0.001
Stroke, n (%)	0 (0.0)	4 (8.0)	4 (8.3)	0.115
Dementia, n (%)	0 (0.0)	1 (2.0)	3 (6.2)	0.151
Cancer, n (%)	0 (0.0)	12 (24.0)^a^	4 (8.3)^b^	0.001

Data are shown as the mean (standard deviation) or n (%). P values were derived from one-way ANOVA, Kruskal-Wallis test or χ^2^ test.

BMI, body mass index; HbA1c, glycated hemoglobin; ASO, arteriosclerosis obliterans

^a^Significantly different compared with the healthy subjects group, *P* < .05

^b^Significantly different compared with the patients without DPN group, *P* < .05

**Table 2:** Physical data

	healthy subjects	patients without DPN	patients with DPN	*P* value	
n	50	50	48		
Grip strength, kg	30.0(10.5)	30.2(10.0)	28.2(9.7)	0.737	
Grip strength/weight, %	52.5(12.9)	44.2(12.0)^a^	42.9(13.3)^a^	< 0.001	
Toe Grip Strength, kg	14.7(8.7)	13.5(6.1)	9.6(4.7)^ab^	< 0.001	
Toe Grip Strength/weight, %	25.4(12.6)	19.8(8.4)^a^	14.5(6.9)^ab^	< 0.001	
Gait speed, m/sec	1.6(0.3)	1.0(0.2)^a^	1.0(0.2)^ab^	< 0.001	
LOS, n (%)	0 (0.0)	5 (10.0)^a^	20 (41.7)^ab^	< 0.001	
VPT, n (%)	0 (0.0)	13 (26.0)^a^	29 (60.4)^ab^	< 0.001	
Sensory symptoms, n (%)	3 (6.0)	17 (34.0)^a^	28 (58.3)^ab^	< 0.001	
Faller, n (%)	2 (4.1)	9 (18.0)	14 (29.8)^a^	0.004	

Data are shown as the mean (standard deviation) or n (%).

P values were derived from one-way ANOVA, Kruskal-Wallis test or χ^2^ test.

LOS, loss of protective sensation; VPT, vibration perception threshold.

^a^Significantly different compared with the healthy subjects group, *P* < .05

^b^Significantly different compared with the patients without DPN group, *P* < .05

Hierarchical multiple regression analysis was used to check whether age, T2DM, and DPN were associated with TGS and TGS/Wt% after controlling for other factors (Table 3). Regression analysis showed an association for age (β = − 0.339), T2DM (β = − 0.162), and DPN (β = − 0.396) in model 2, controlling for TGS confounding factors. In model 4, controlling for TGS/Wt% confounders, associations were also found for age (β = − 0.293), T2DM (β = − 0.273), and DPN (β = − 0.491). The adjusted multiple correlation coefficient squared (R^2^) of the model considering confounding factors was 0.374 for model 2 and 0.320 for model 4. This indicates that the enumerated factors explained 37% of the variance in TGS and 32% in TGS/Wt%. The variance inflation factor was 1.084–1.567 for model 2 and 1.054–1.392 for model 4, indicating no significant multicollinearity. The Durbin-Watson ratio was 1.972 for model 2 and 1.871 for model 4. No autocorrelation was found in the samples.

**Table 3:** Results of multiple regression analysis

	Independent value	B	95% CI	$\beta$	*P* value	ANOVA	R^2^	
TGS								
Model 1	Age	− 0.223	− 0.30, − 0.15	− 0.422	< 0.001	< 0.001	0.256	
	T2DM	− 1.059	− 3.46, 1.34	− 0.071	0.385			
	DPN	− 4.304	− 6.75, − 1.86	− 0.287	0.001			
Model 2	Age	− 0.179	− 0.25, − 0.11	− 0.339	< 0.001	< 0.001	0.374	
	T2DM	− 2.402	− 4.80, − 0.01	− 0.162	0.049			
	DPN	− 5.942	− 8.33, − 3.55	− 0.396	< 0.001			
	Sex	− 5.107	− 7.00, − 3.22	− 0.363	< 0.001			
	BMI	0.148	− 0.07, 0.36	0.097	0.177			
TGS/Wt (%)								
Model 3	Age	− 0.268	− 0.38, − 0.16	− 0.337	< 0.001	< 0.001	0.274	
	T2DM	− 5.381	− 8.95, − 1.82	− 0.241	0.003			
	DPN	− 9.931	− 13.56, − 6.31	− 0.441	< 0.001			
Model 4	Age	− 0.233	− 0.34, − 0.12	− 0.293	< 0.001	< 0.001	0.320	
	T2DM	− 6.090	− 9.57, − 2.61	− 0.273	< 0.001			
	DPN	− 11.055	− 14.63, − 7.48	− 0.491	< 0.001			
	Sex	− 4.852	− 7.79, − 1.91	− 0.230	< 0.001			

B, partial regression coefficient; 95% CI, 95% confidence interval; ${\upbeta }$, standardized partial regression coefficient;

ANOVA, analysis of variance; R^2^, multiple correlation coefficient adjusted for the degrees of freedom; TGS, toe grip strength; TGS/Wt, TGS-to-weight ratio; BMI, body mass index; T2DM, type 2 diabetes mellitus; DPN, Diabetic polyneuropathy

## Discussion

This study investigated the factors associated with TGS in patients with and without DPN and healthy subjects. In the three-group comparison, the patients with DPN had the most significant muscle weakness, based on both TGS and TGS/Wt%. Patients with DPN also had a higher fall rate than healthy subjects. On hierarchical multiple regression analysis with TGS and TGS/Wt% as dependent variables, age, T2DM, and DPN were still identified as factors after adjustment for sex and BMI. This is the first study to show that older age and the presence of T2DM and DPN were the most significant factors related to TGS. It is generally known that most patients with T2DM are obese, with large muscle mass and high body fat content. Therefore, a previous study pointed out that comparing muscle strength without adjustment for muscle mass and body fat mass can be misleading [26]. In the present study, both TGS and TGS/Wt% accounting for each individual weight were calculated. In addition, the results of the present study showed that age was identified as a significant factor related to TGS. Previous study investigating toe flexor muscle mass by ultrasound reported a 19–49% decrease in muscle cross-sectional area and a 32% decrease in TGS due to aging [27, 28]. Aging causes cellular and molecular changes that result in muscle weakness [29] and age-related changes in muscle fiber composition [30], which may have been a factor associated with TGS.

T2DM was a factor related to TGS in the present study. In a previous study comparing knee extensor muscle strength between patients with T2DM and healthy subjects, a significant reduction in muscle strength was observed in patients with T2DM, similar to the present study [26]. The onset of T2DM in older people has been reported to accelerate the decrease in muscle function and strength [31]. On the other hand, a recent study reported that the presence of T2DM alone does not affect muscle strength or muscle mass when compared to patients with T2DM and healthy subjects [32]. In comparisons between patients with T2DM and healthy subjects, it has also been reported that muscle weakness is more common in distal than in proximal muscles of the lower limb [8]. Hence, in the present study, patients with T2DM may have shown muscle weakness because muscle strength was evaluated by TGS in the distal lower limb, not by knee extension strength in the proximal lower limb.

A cross-sectional study comparing knee extensor strength in patients with T2DM reported significantly lower knee extensor strength in patients with DPN [6]. In the present study, the results support the previous studies’ finding that the presence of DPN is associated with muscle weakness. The cross-sectional studies using MRI have reported that DPN is associated with reduced muscle mass [33] and increased intramuscular fat [32] compared with those without DPN. A previous cross-sectional study using foot muscle strength as an outcome, which included only male patients with T2DM, reported that toe pinch force was reduced by having DPN [14]. However, it should be noted that the study subjects were only males and that adjustment for confounding factors was insufficient, with adjustment only for age and BMI. The present study investigated factors related to TGS, including sex differences. The present study also used nerve conduction testing to accurately and objectively identify DPN. Most studies of muscle strength in patients with T2DM have focused on knee extensor strength. However, TGS assessment is essential in T2DM, where the distal muscles are more affected than the proximal muscles of the lower limb. In addition, the fall rate was increased in the patients with DPN group compared with the healthy subjects in the present study.

Aerobic exercise, such as walking, is recommended for diabetic patients as part of exercise therapies for DM [34]. To perform aerobic exercise safely to manage DM, TGS assessment may help to determine the risk of falls. Meanwhile, DPN and foot muscle reduction have been reported to be associated with diabetic foot [35], and diabetic foot may result in lower limb amputation; thereby, diabetic foot is associated with decreased health-related quality of life [36]. Consequently, providing proper foot care for patients with T2DM in necessary. Furthermore, nerve conduction studies are time- and equipment-intensive tests and are often difficult to perform in day-to-day clinical practice. In the future, if the thresholds for T2DM and DPN are determined by the simple TGS measurement used in this study, it will be helpful in daily clinical practice for fall prevention and foot care in patients with T2DM.

A limitation of this study is that patients were divided into two groups using the Baba classification, one with and one without neuropathy, although the Baba classification is classified into five levels in the original method. Future studies should focus on the severity of diabetic neuropathy. In addition, nerve conduction test results were not available in healthy subjects. However, there were no positive controls in the healthy subjects for the LOPS and vibratory perception tests. Therefore, the influence on the results of this study was considered minimal. The present study design does not clarify the relationship between TGS and falls in patients with T2DM. A systematic review of fall prevention by increasing toe flexor muscle strength found that TGS strengthening exercises improved dynamic balance in a study of young healthy people [37]. However, the relationship between TGS and falls in patients with T2DM is not fully understood. In the future, prospective studies should be conducted to clarify the relationship between TGS and falls and the effect of fall prevention on TGS.

## Conclusion

The results of this study suggest that age, T2DM, and DPN should be considered factors related to TGS in clinical practice. These factors should be considered when providing fall prevention, foot care, and rehabilitation for patients with T2DM.
